# Multiple System Atrophy Masquerading as Drug-Resistant Orthostatic Hypotension

**DOI:** 10.7759/cureus.69774

**Published:** 2024-09-20

**Authors:** Manami Yamaoka, Shuichiro Neshige, Kenta Sasaki, Hirofumi Maruyama

**Affiliations:** 1 Department of Clinical Neuroscience and Therapeutics, Hiroshima University Graduate School of Biomedical and Health Sciences, Hiroshima, JPN

**Keywords:** cerebellar-ataxia, msa-multiple system atrophy, neurogenic orthostatic hypotension, parkinson-plus syndromes, parkinson's disease

## Abstract

A 54-year-old male presented with a two-year history of recurrent syncope. Although he was treated with droxidopa and amezinium metilsulfate, his syncope was drug-resistant. Blood tests, cardiac evaluation and brain computed tomography (CT) were unremarkable. As his Schellong test was positive, orthostatic hypotension and syncope were the tentative diagnoses. Upon evaluating the patient neurologically while carefully avoiding syncope induction, mild cerebellar ataxia was observed. However, a brain magnetic resonance imaging (MRI) scan revealed a possible "cross sign" in the pons. Subsequent dopamine transporter single-photon emission computed tomography (DAT-SPECT) showed reduced uptake in the basal ganglia. Based on these findings, a clinical diagnosis of probable multiple system atrophy of the cerebellar type (MSA-C) was made, and treatment with taltirelin hydrate was initiated. This case underscores the importance of considering multiple system atrophy (MSA) in patients with persistent orthostatic hypotension and syncope, even before more obvious neurodegenerative symptoms emerge.

## Introduction

Orthostatic hypotension, characterized by a drop in blood pressure upon standing, leads to dizziness and fainting [1]. It can be classified into primary and secondary causes [2]. Primary orthostatic hypotension results from autonomic nervous system dysfunction, while secondary causes include medications, dehydration, and endocrine disorders. The persistence of autonomic dysfunction despite lifestyle modification and drug therapy should be searched for as a cause of neurodegenerative diseases such as multiple system atrophy (MSA) [3]. This report presents a case of MSA where syncope from autonomic dysfunction appeared before more obvious symptoms of cerebellar ataxia and parkinsonism. Additionally, because autonomic neuropathy restricted his daily activities due to the fear of fainting and reduced his quality of life, there was delay to realize that his cerebellar damage was progressing slowly.

## Case presentation

A 54-year-old male presented to the emergency room. He complained of "a wobbly feeling when standing up that did not improve," which prompted a referral to our department for drug-resistant orthostatic hypotension evaluation. This episode occurred when he stood up after lunch, causing him to lose consciousness and fall. Diagnosed with orthostatic hypotension, he was observed but continued to experience syncope with positional changes. He was prescribed droxidopa and amezinium metilsulfate, but the syncope persisted, leading to fear of standing and reduced walking. The patient had no history of excessive alcohol use, and his resting vital signs were normal except for high blood pressure. However, the Schellong test demonstrated a drop in systolic blood pressure by 47 mmHg (from 145 mmHg to 98 mmHg), accompanied by a feeling of "fainting" that remained uncompensated and ceased immediately after he sat down. During the interview, frequent urination and constipation were reported. Despite slow positional movements due to fear of syncope, a detailed neurological assessment revealed mild cerebellar ataxia. On neurological examination nystagmus was negligible. No dysmetria was noted on the finger-to-nose test; however, a terminal tremor was present (Table 1).

**Table 1:** Patient's neurological findings Neurological findings show mild cerebellar ataxia

Parameters	Findings
Eye movements	Overshoot
Dysarthria	Mild slurred
Finger-nose-finger test	Terminal tremor
Tendon reflex	Accelerate
Romberg test	Negative
Mann posture	Unstable

Physical examination showed no heart murmurs or carotid bruits. Thus, our differential diagnoses based on the history and physical examination of the patient before ordering investigations were Parkinson's disease, amyloidosis, autoimmune autonomic ganglionopathy, and alcoholic cerebellar ataxia.

**Figure 1:**
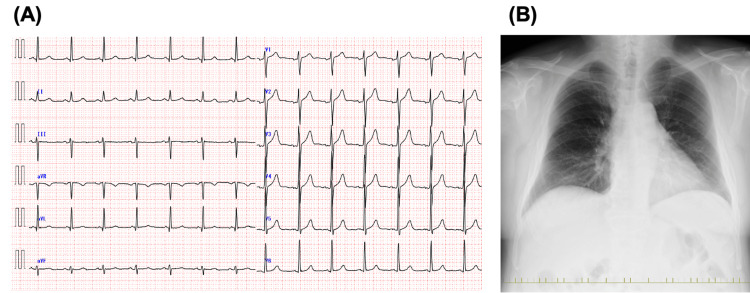
Electrocardiogram (ECG) and chest X-ray. (A) ECG reveals a sinus rhythm at 82 bpm with normal intervals. (B) The chest X-ray shows normal findings, with a cardiothoracic ratio of 50%.

The electrocardiogram and chest X-ray were normal (Figure 1).

Blood tests ruled out diabetes and thyroid dysfunction (Table 2). Furthermore, anti-ganglionic acetylcholine receptor antibodies were negative.

**Table 2:** The studied laboratory parameters along with the normal range for reference. The results of the blood tests are shown. WBC: white blood cells, RBC: red blood cells, Hb: hemoglobin, PLT: platelet, T-Bil: total bilirubin, AST: aspartate aminotransferase, ALT: alanine aminotransferase, LD: lactate dehydrogenase, IFCC: International Federation of Clinical Chemistry and Laboratory Medicine, CK: creatine kinase, AMY: amylase, Na: sodium, K: potassium, Cl: chloride, UN: urea nitrogen, CRE: creatinine, HbA1c: glycosylated hemoglobin, Glu: glucose, F-T4: free thyroxine. TSH: thyroid-stimulating hormone.

Parameters	Values	Reference range
WBC (×10^3/μL)	9.27	3.3-8.6
RBC (×10^6/μL)	4.18	4.35-5.55
Hb (g/dL)	12.6	13.7-16.8
PLT (×10^3/μL)	203	158-348
T-Bil (mg/dL)	1.1	0.4-1.5
AST (U/L)	30	13-30
ALT (U/L)	61	10-42
LD (IFCC) (U/L)	147	124-222
CK (U/L)	57	59-248
AMY (U/L)	42	44-132
Na (mmol/L)	140	138-145
K (mmol/L)	4.1	3.6-4.8
Cl (mmol/L)	106	101-108
UN (mg/dL)	23.8	8.0-20.0
CRE (mg/dL)	0.97	0.65-1.07
HbA1c (%)	5.5	4.9-6
Glu (mg/dL)	114	73-109
Folic acid (ng/ml)	4.9	3.9-26.8
Vitamin B12 (pg/mL)	502	197-771
F-T4 (ng/dL)	1.3	1.1-1.8
TSH (μIU/mL)	0.354	0.5-5.0
Ratio of κ to λ	1.31	0.26-1.65

**Figure 2:**
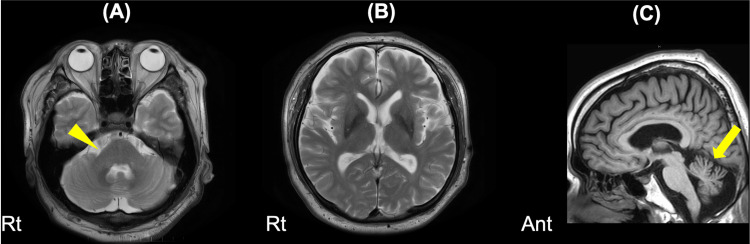
MRI with contrast showing cross sign and cerebellar atrophy. (A) T2-weighted images on axial views are shown. The yellow arrowhead indicates a possible finding of a cross sign in the pons. (B) Atrophy of the putamen and linear T2 hyperintensity in the lateral portion of the putamen were not observed. (C) T1-weighted image on sagittal view is shown. Cerebellar atrophy may be present (yellow arrow).

Magnetic resonance imaging (MRI) with contrast showed no cerebrovascular region but suggested a possible cross sign in the pons (Figure 2). This sign is characteristic of MSA.

**Figure 3:**
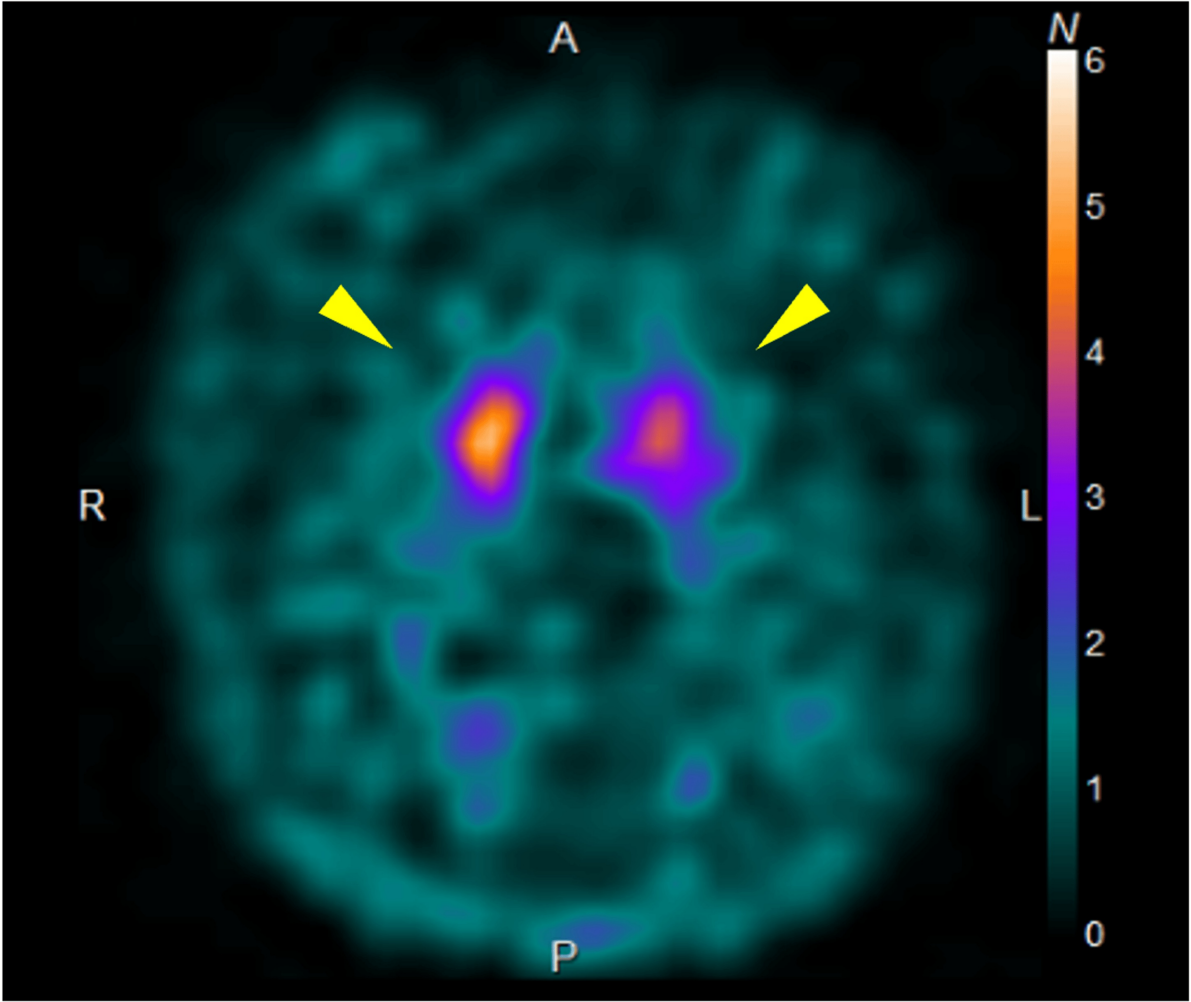
DAT-SPECT image showing reduced uptake. Yellow arrowhead indicates reduced uptake which is shown in both basal ganglia, predominantly on the left. The specific binding ratio (SBR) is 7.59 on the right and 6.09 on the left. DAT-SPECT: dopamine transporter single-photon emission computed tomography.

Dopamine transporter single-photon emission computed tomography (DAT-SPECT) showed reduced uptake in both basal ganglia (Figure 3). 

Based on these findings, a clinical diagnosis of probable multiple system atrophy of the cerebellar type (MSA-C) was made, and treatment with taltirelin hydrate was initiated [4]. At the two-month follow-up visit, the physical and neurological examination findings were unchanged.

## Discussion

This case report discusses a patient with MSA-C where orthostatic hypotension from autonomic dysfunction appeared before more prominent cerebellar ataxia. The patient exhibited drug-resistant orthostatic hypotension, and his fear of syncope led to reduced activity, masking the identification of cerebellar ataxia that was progressing gradually. Despite experiencing unsteadiness during positional changes, the present patient was unable to distinguish between the "feeling of faintness" due to orthostatic hypotension and the "feeling of lightheadedness' due to cerebellar ataxia," complicating early detection of cerebellar ataxia by non-specialists. Thus, in cases of drug-resistant orthostatic hypotension, neurodegenerative diseases like MSA should be considered [5].

Syncope is characterized by rapid onset, short duration, and spontaneous complete recovery. Though transient, syncope can lead to injuries from falls, making cause identification and recurrence prevention crucial. Syncope etiology includes orthostatic hypotension, neurally mediated syncope, and cardiac syncope. Orthostatic hypotension refers to a significant drop in blood pressure upon standing, often due to autonomic dysfunction, Parkinson's disease, diabetic autonomic neuropathy, or reduced blood volume from dehydration, diarrhea, or bleeding [6-8]. Neurally mediated syncope results from transient cerebral hypoperfusion due to sympathetic inhibition and vagal activation, while cardiac syncope is caused by arrhythmias or structural heart disease.

Autonomic dysfunction is a hallmark of MSA and can present early [9]. Specific examples other than syncope include urinary dysfunction, gastrointestinal issues (constipation, gastroparesis, dysphagia), sexual dysfunction (erectile dysfunction, decreased libido), and sweating abnormalities. When syncope from autonomic dysfunction is observed with these symptoms, MSA should be considered, and further evaluation for cerebellar symptoms or parkinsonism should be conducted for early diagnosis. Early diagnosis can be facilitated by imaging studies, including identifying the cross sign in the pons on MRI, lateral putaminal atrophy in both basal ganglia, and decreased uptake on DAT-SPECT [10,11]. Conversely, as we evaluated in the present case, autoimmune autonomic ganglionopathy should also be considered as a differential diagnosis [12].

## Conclusions

In conclusion, in cases of drug-resistant orthostatic hypotension due to autonomic dysfunction, it is essential to be alert for the emergence of neurological conditions such as cerebellar ataxia or Parkinson's disease. It is also important to consider MSA in patients who present with persistent orthostatic hypotension and syncope, even before the appearance of more obvious neurodegenerative symptoms. Early diagnosis is facilitated by imaging studies such as brain MRI and DAT-SPECT, as well as a thorough symptom evaluation.
